# Do First Ray-Related Angles Change following Subtalar Arthroereisis in Pediatric Patients? A Radiographic Study

**DOI:** 10.3390/children11070760

**Published:** 2024-06-22

**Authors:** Antonio Mazzotti, Laura Langone, Simone Ottavio Zielli, Elena Artioli, Alberto Arceri, Lorenzo Brognara, Francesco Traina, Cesare Faldini

**Affiliations:** 1 1st Orthopaedic and Traumatology Clinic, IRCCS Istituto Ortopedico Rizzoli, 40136 Bologna, Italy; antonio.mazzotti@ior.it (A.M.); laura.langone@ior.it (L.L.); elena.artioli@ior.it (E.A.); alberto.arceri@ior.it (A.A.); cesare.faldini@ior.it (C.F.); 2 Department of Biomedical and Neuromotor Sciences, University of Bologna, 40126 Bologna, Italy; lorenzo.brognara2@unibo.it (L.B.); francesco.traina2@unibo.it (F.T.); 3 Orthopedics and Traumatology, Prosthetic, and Reimplantation Surgery of the Hip and Knee Clinic, IRCCS Istituto Ortopedico Rizzoli, 40136 Bologna, Italy

**Keywords:** subtalar arthrorisis, pediatric, flatfoot, hallux valgus angle, intermetatarsal angle

## Abstract

Introduction: Subtalar Arthroereisis (STA) is a surgical intervention for pediatric flexible flatfoot (PFF), primarily targeting hindfoot alignment by limiting excessive subtalar eversion. However, its effects on forefoot parameters remain underexplored. This study aims to investigate radiological changes following STA in pediatric patients. Materials and Methods: A retrospective analysis was conducted on consecutive patients treated with STA for PFF. First ray-related angles, including the Hallux Valgus Angle (HVA) and the Intermetatarsal Angle (IMA), alongside hindfoot radiological parameters such as the Meary, Calcaneal Pitch, and Costa Bartani angles, were assessed. Subgroup analysis by gender was performed, and correlations between demographic and preoperative radiological parameters were examined. Results: Forty-one patients (81 feet) with an average age of 11.6 years were included, with a mean follow-up duration of 6.4 months. No significant differences were observed in first ray-related angles pre-and postoperatively, with the mean IMA changing from 7.97° to 7.18° and the mean HV angles changing from 9.51° to 8.66°. Noteworthy improvements were seen in flat foot angles, including the Meary, Calcaneal Pitch, and Costa Bartani angles, postoperatively. The age subgroup analysis revealed similar trends in IMA and HVA changes between Group A (who underwent surgery before peak growth) and Group B (who underwent surgery after peak growth). Higher preoperative angles tended to improve, while lower preoperative IMAs and HVAs tended to worsen postoperatively, all remaining within normal ranges. Conclusion: STA showed positive radiological outcomes for PFF treatment, while negligible changes in first ray-related angles were observed. The age subgroup analysis indicated similar trends regardless of operation timing. Higher preoperative angles tended to improve, while lower preoperative angles tended to worsen postoperatively, despite all falling within non-pathological ranges. Further research is warranted to confirm this correlation.

## 1. Introduction

Subtalar Arthroereisis (STA) is among the surgical options for pediatric flexible flatfoot (PFF), offering various techniques and implant choices [1]. The fundamental principle of STA involves limiting excessive subtalar eversion through the insertion of a device into the sinus tarsi, thereby preserving the natural alignment between the talus and calcaneus during bone remodeling [2,3].

Subtalar eversion is associated with forefoot deformities, such as Hallux Valgus, while PFF, characterized by hindfoot pronation during walking, disrupts the midtarsal joint locking, leading to a larger Intermetatarsal Angle (IMA) and increasing instability in the first metatarsophalangeal joint [4].

Despite ongoing debates regarding the optimal management of PFF in children, numerous studies have documented satisfactory radiographic and clinical outcomes, along with high patient satisfaction rates, following STA [5]. Existing studies primarily focus on the radiographic parameters of the hindfoot, overlooking potential changes in other areas. Given the influence of the subtalar joint on the midfoot and forefoot, it is plausible that correcting a flatfoot deformity may result in changes in forefoot parameters, particularly at the first ray. While previous studies in adults have investigated radiographic changes in the first ray following flatfoot deformity correction [6,7], with controversial results, no similar investigations have been conducted in pediatric patients with flexible flatfoot.

This study aims to investigate whether there are changes in radiological parameters associated with first-ray deformity assessment following surgical correction of PFF using STA, focusing specifically on measuring the HVA and IMA.

## 2. Materials and Methods

### 2.1. Study Design

A retrospective cross-sectional study was performed on patients who received STA with bioabsorbable polymeric endo-orthotic implants for symptomatic PFF. The study adhered to the guidelines of the Declaration of Helsinki [8].

### 2.2. Patients

Inclusion criteria encompassed individuals who underwent STA with a bioabsorbable polymeric endo-orthotic implant (BFFI^®^ Novagenit S.R.L., Mezzolombardo, Italy) between April 2021 and March 2023 at a single institution. Eligible patients were aged between eight and fifteen years at the time of surgery, presenting symptomatic idiopathic PFF as the primary indication.

Exclusion criteria included rigid flatfoot, posttraumatic flatfoot, clubfoot sequelae, generalized joint hyperlaxity, neurogenic or neuromuscular disorders, comorbidities, history of lower limb surgeries, Achille’s tendon lengthening (ATL), and incomplete radiographic assessments.

Patients who underwent ATL were excluded because of the potential changes in radiographic forefoot value following this procedure previously described [9]. 

The institutional database was systematically searched to identify eligible patients.

### 2.3. Implant Characteristics, Operative Technique, and Post-Operative Protocol

The utilized implant (BFFI^®^ Novagenit SRL, Mezzolombardo (TN), Italy) was a bioabsorbable Poly-L-lactic acid (PLLA) endo-orthotic extra-articular device, classified as extraosseous sinus tarsi stents (EOTTS) [2]. The implant is composed of two parts: an external cylindrical body (plug) and an inner cylindrical body (screw). The surgical procedure adhered to the previously reported method [10]: a 1 cm incision was performed on the skin overlying the distal–central part of the sinus tarsi. Specialized blunt rods, gradually increasing in size, were inserted in the identical direction to prepare the sinus tarsi for implant insertion. The diameter of the largest rod corresponded to the ideal size of the implant. Two small retractors were utilized to open the skin and retinaculum fibers, aiding in the placement of the outer cylinder of the implant using a universal introducer. Subsequently, the inner screw was inserted to expand and secure the implant. 

Postoperatively, the feet were immobilized with a cast for the initial 4 weeks: no weight bearing was permitted for the first 2 weeks, followed by progressive weight bearing over the subsequent 2 weeks. After 4 weeks, casts were removed, and patients were permitted to walk with regular shoes. The postoperative protocol advised against high-risk activities, jumping, and running for the first 5–6 months while permitting swimming and stationary biking. Pediatric patients undergoing STA are typically capable of fully returning to sports activities in most cases after 6 months. Consequently, it could be assumed that at this point the condition can be regarded as sufficiently stable to be assessed with weight-bearing radiographs without any abnormalities in foot support. [11].

### 2.4. Outcomes

The primary objective was to assess significant differences in forefoot radiological parameters pre- and post-STA. HVA and IMA were measured on preoperative and 6-month follow-up radiographs under full weight-bearing conditions. Normal ranges for the parameters were defined as follows: HVA angles < 15° [12]; IMA angles < 9° [12].

The secondary aim was to observe hindfoot radiological parameter modifications after STA, measuring Meary’s Angle (MA), Calcaneal Pitch angle (CP), and Moreau Costa Bertani angle (MCB) on preoperative and 6-month follow-up radiographs. Normal ranges for the parameters were defined as follows: MA [13] between −4° and +4°; CP [13] angle between 18 and 20°; MCB [14] angle between 115 and 125°.

The tertiary objective was to investigate any possible correlations between angular variations in the first ray and preoperative patients’ characteristics. Patients were divided into two groups based on the timing of their surgery relative to peak growth. This classification was based on the literature which indicated that peak growth velocity occurs at 11.5 years in females and 13.5 years in males [15]. Patients were classified as Group A if they underwent surgery before reaching peak growth, and as Group B if they had the surgery afterward.

Measurements were conducted on preoperative and 6-month follow-up radiographs by two independent orthopedic surgeons, and interobserver reliability was evaluated. All measurements were performed on calibrated images with the PACS^®^ system (GE-Healthcare, Chicago, IL, USA).

Six months was considered the minimum time to deem the condition stabilized and to allow weight-bearing without pain, as patients can typically resume sports activities at this time.

### 2.5. Statistics

The statistical unit in this study was represented by each individually operated foot. A post hoc power analysis was conducted to ensure a sufficient sample size for detecting clinically relevant differences between pre- and postoperative radiographic values. Assuming a significant difference in HVA measurement requires a minimum 5-degree difference [16], and in IMA it measurement requires a minimum of 3.60 degrees [17] of difference between two measurements; a sample size of at least 70 feet was calculated.

Intra-observer reliability was assessed using intraclass correlation coefficients (ICCs) for continuous data, with a Cronbach’s alpha exceeding 0.80 signifying excellent inter-observer agreement. Cohen’s kappa coefficient was calculated to measure inter-observer reliability. Variables resulting from radiological scores were reported as mean values, standard deviations (SD), and ranges. 

The normality of the distribution for continuous variables was assessed using the Shapiro–Wilk test. After confirming normal distribution to assess whether the mean difference between pre- and postoperative angular values exceeded the literature-recommended threshold, a *t*-test was applied with a decision criterion based on the confidence interval for the difference between means at *p* < 0.05 [16,17].

Any potential correlation between the two variables was assessed using Pearson’s test. Local estimated scatterplot smoothing was performed to visualize the correlation between the changes in radiological parameters between pre- and post-operation and the preoperative deformity or the age of the patients at the time of surgery.

Statistical significance was defined as *p* < 0.05, and all statistical analyses were performed using Jamovi software (The Jamovi project—jamovi Version 1.6, 2021).

## 3. Results

Forty-one patients, corresponding to 81 feet, were identified and included in the study. Among these patients, fourteen (34.1%) were females. The mean age of the population at the time of surgery was 11.6 ± 1.71 years, ranging from 8 to 15 years. Demographic details of the patients are presented in Table 1. All patients were treated by the same surgeon.

The average follow-up duration for radiological assessment was 6 months. Subgroup analysis was conducted, considering patients’ gender, age, and growth potential at the time of surgery.

No intraoperative complications occurred. Regarding postoperative complications, three children experienced delayed wound healing, which was resolved within a week following cast removal with no residual effects. Additionally, five children reported residual pain after cast removal, which had completely disappeared at the 8-week follow-up examination. There was no need for revision surgery among the patients. A total of forty out of the forty-one patients (97.5%) underwent a single-stage bilateral procedure, while only one patient (2.5%) had surgery exclusively on the right foot.

Intra-observer reliability showed excellent values, with an ICC of 0.91 (CI 0.87–0.93). Inter-observer reliability was also excellent with a Cohen’s kappa coefficient of 0.89 (CI 0.85–0.92). 

Concerning the measurements of first ray-related angles, no significant difference was observed between the preoperative and postoperative measurements (see Table 1 and Table 2).

**Figure 1:**
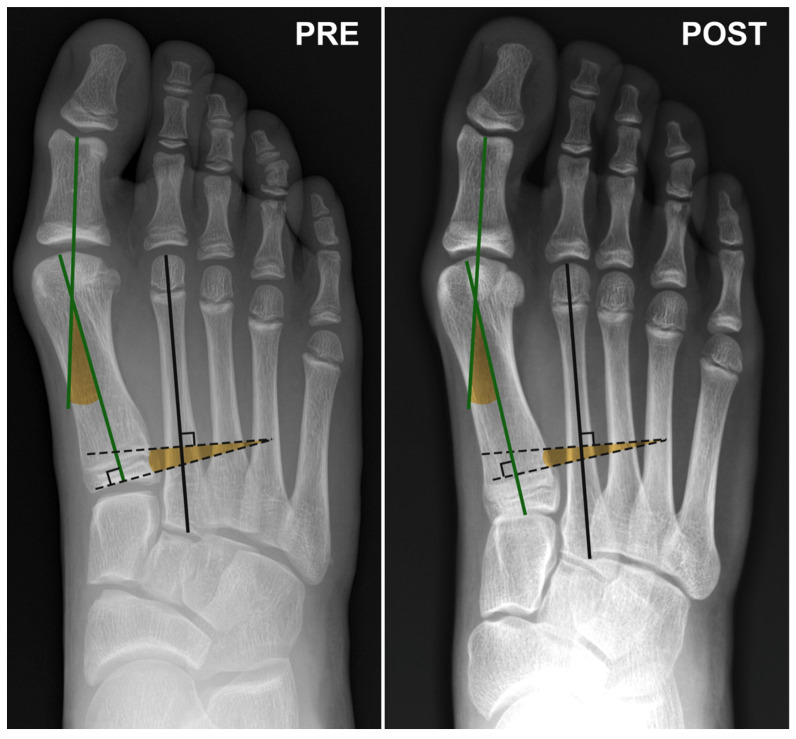
Dorsoplantar X-ray view of the foot of an 11-year-old female patient at the time of surgery and at the 6-month postoperative follow-up. Angle subtended by the green lines: Hallux Valgus Angle (HVA); Angle subtended by the black dashed lines: InterMetatarsal Angle (IMA).

**Figure 2:**
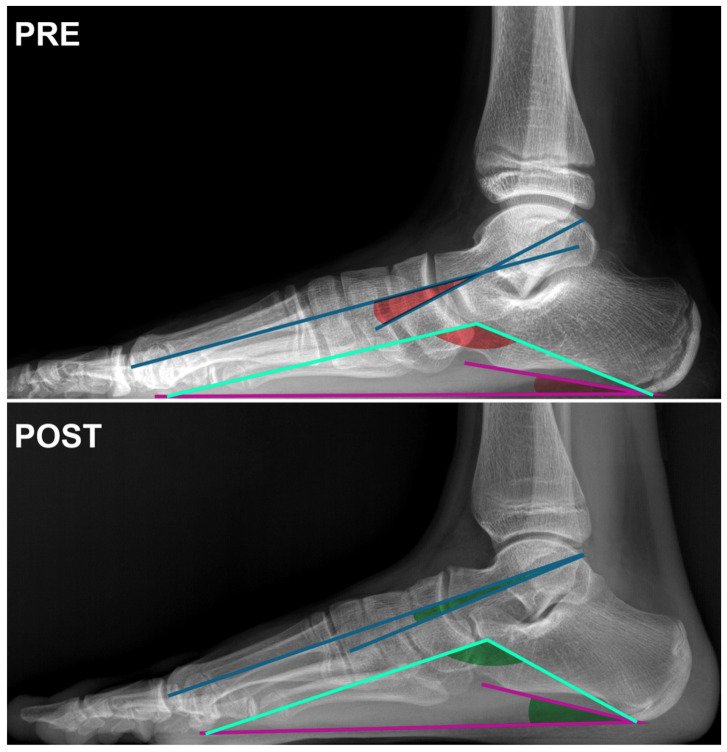
Lateral X-ray view of the foot of an 11-year-old female patient at the time of surgery (PRE) and at the 6-month postoperative follow-up (POST). Angle subtended by the blue lines: Meary’s angle; angle subtended by the cyan lines: Moreau-Bartani’s angle; angle subtended by the purple lines: Calcaneal Pitch angle.

Regarding hindfoot-related angles, Meary, Costa Bartani, and Calcaneal Pitch showed an improvement but no significant difference was observed between the preoperative and postoperative measurements (Table 1 and Table 2, Figure 1 and Figure 2).

**Figure 3:**
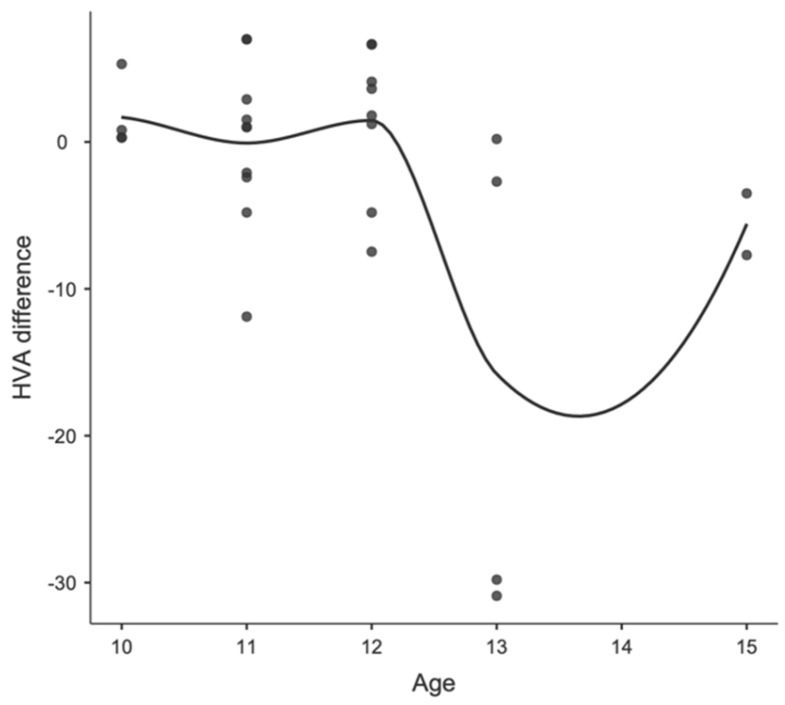
Locally estimated scatterplot smoothing between age and HVA difference in girls.

**Figure 4:**
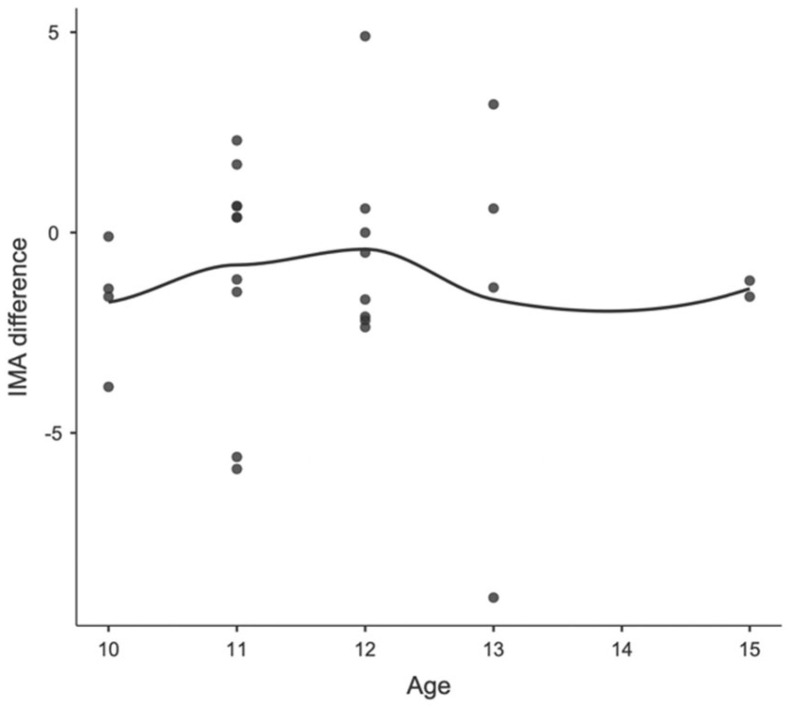
Locally estimated scatterplot smoothing between age and IMA difference in girls.

**Figure 5:**
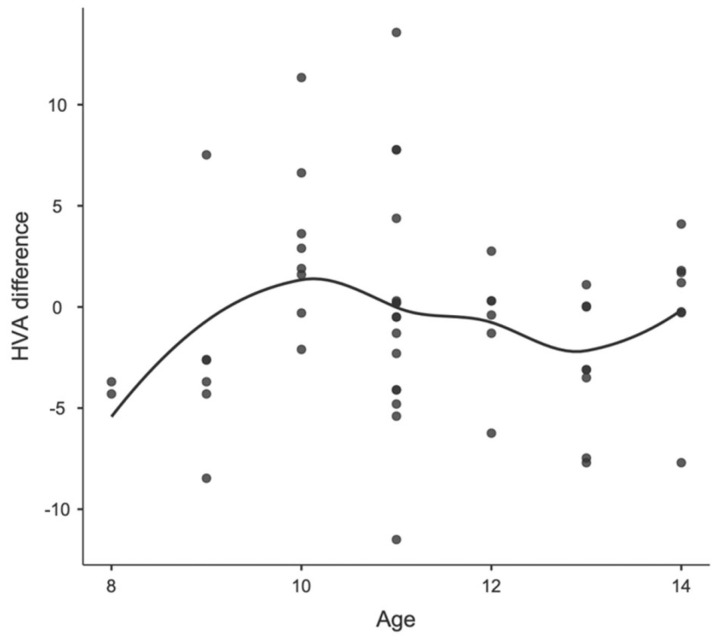
Locally estimated scatterplot smoothing between age and HVA difference in boys.

**Figure 6:**
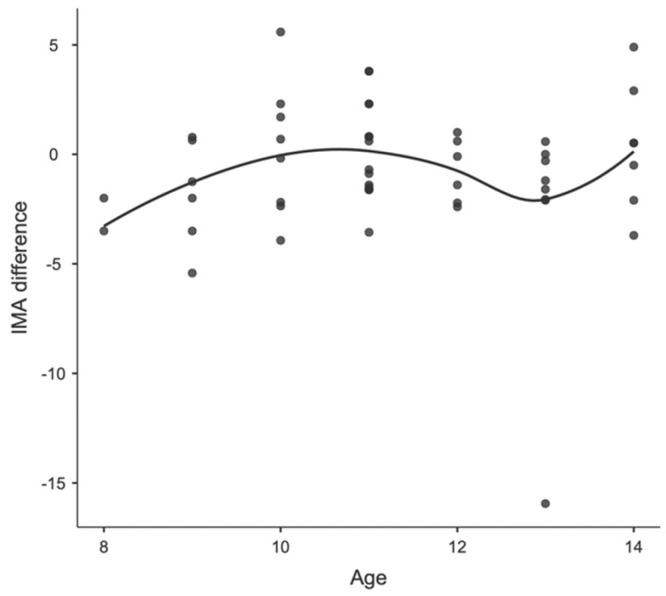
Locally estimated scatterplot smoothing between age and IMA difference in boys.

Concerning age differences, in Group A, including patients before peak growth, the mean IM angle changed from 8.07 degrees (SD 3.04; range 3.10–23.4) to 7.16 degrees (SD 2.16; range 1.50–13.4) and the mean HV angle changed from 8.34 degrees (SD 7.95; range 0.00–47.6) to 8.02 (SD 8.05; range 0.0–42.8). In Group B, including patients after peak growth, the mean IM angle changed from 7.71 degrees (SD 2.13; range 4.80–13.6) to 7.22 degrees (SD 2.51; range 1.10–11.5) and the mean HV angle changed from 12.80 degrees (SD 8.24; range 1.40–31.90) to 10.5 (SD 6.85; range 1.0–22.70). None of these changes were statistically significant. Furthermore, no correlation was found between age and postoperative parameter changes, as observed in the locally estimated scatterplot smoothing (LOESS) plots, in either the male or female patients. The correlations between age, sex, and changes in IMA and HVA are shown in Figure 3 and Figure 4 for girls and Figure 5 and Figure 6 for boys. 

**Figure 7:**
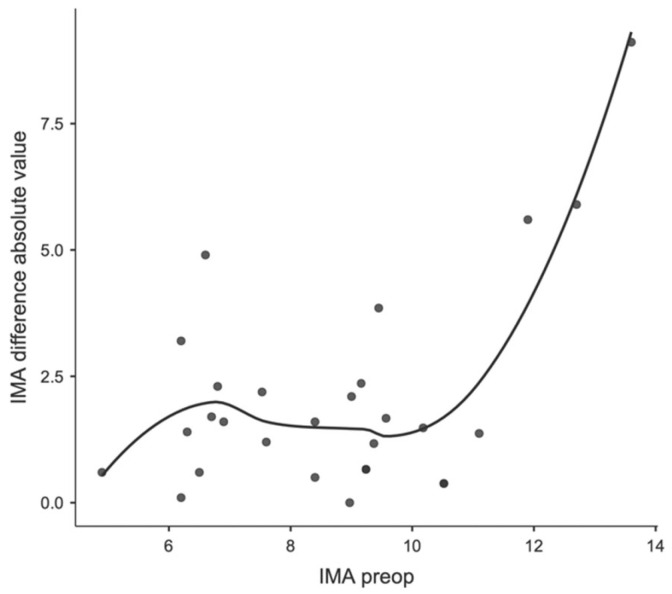
Locally estimated scatterplot smoothing between preoperative IMA and IMA change at the postoperative control, expressed as absolute values.

**Figure 8:**
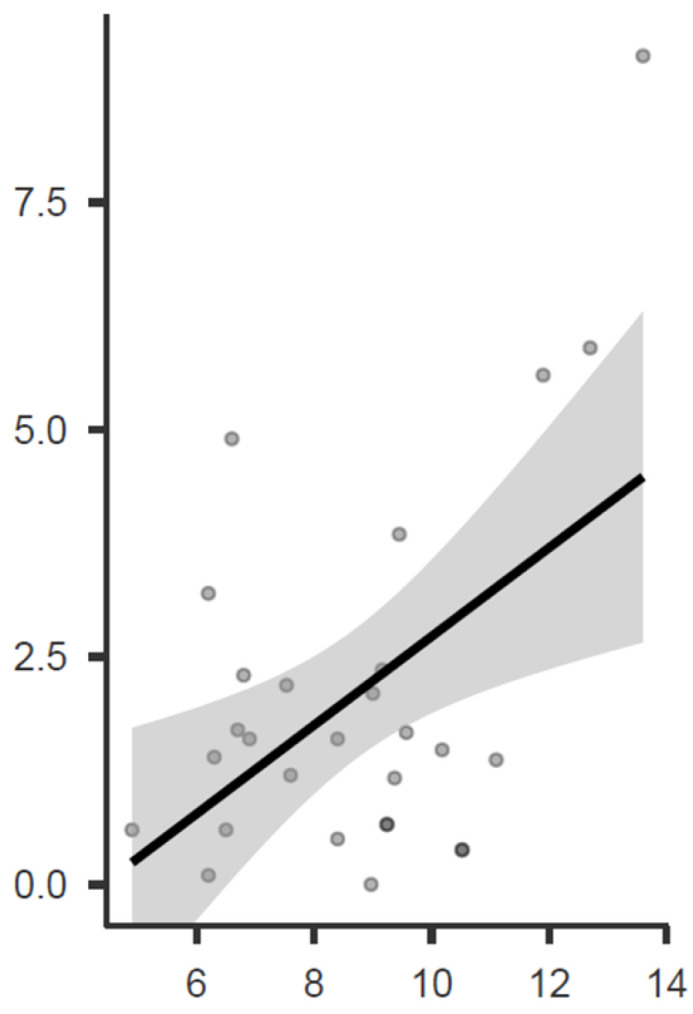
Graphical representation of the Pearson correlation matrix between preoperative IMA and IMA change at the postoperative control, expressed as absolute values.

**Figure 9:**
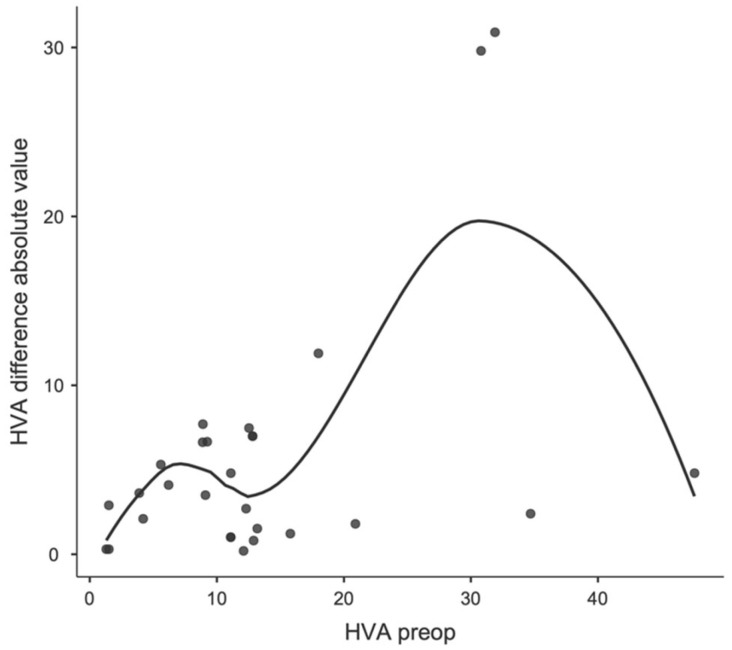
Locally estimated scatterplot smoothing between preoperative HVA and HVA change at the postoperative control, expressed as absolute values.

**Figure 10:**
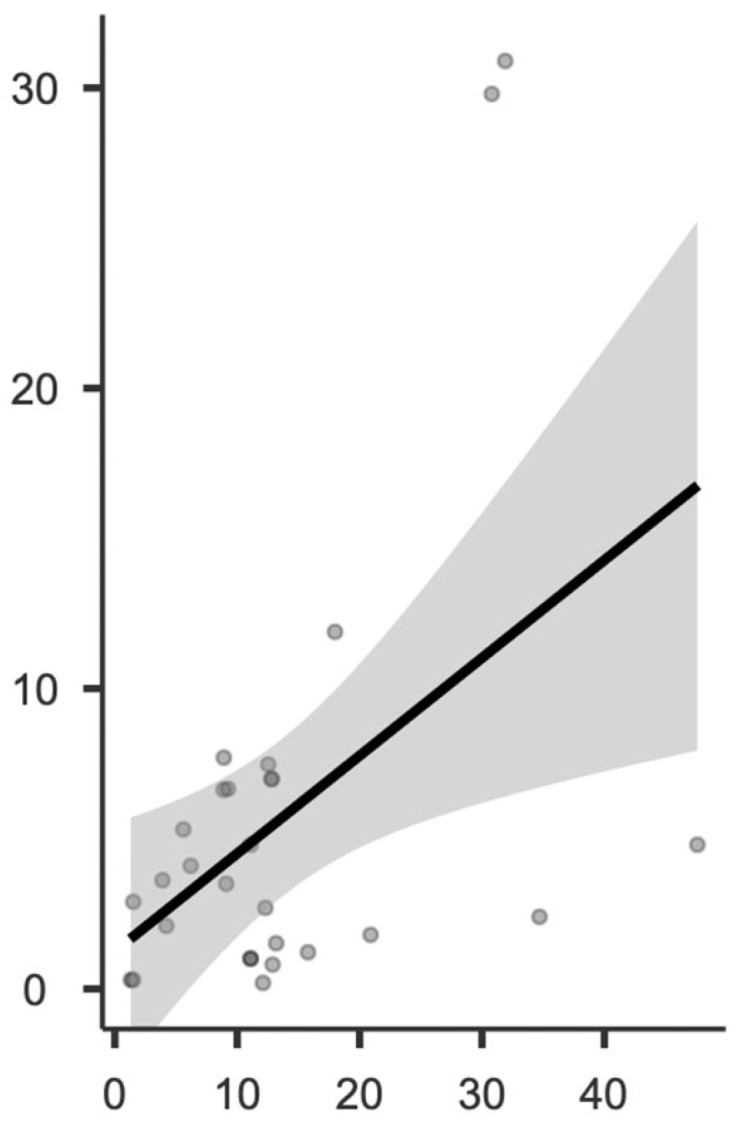
Graphical representation of the Pearson correlation matrix between preoperative HVA and HVA change at the postoperative control, expressed as absolute values.

Regarding a possible correlation between preoperative values and first ray-related angles, a significant correlation was observed between the preoperative IMA and the change in IMA before and after surgery (see Figure 7 and Figure 8), as well as between the preoperative HVA and the change in HVA before and after surgery (Figure 9 and Figure 10).

## 4. Discussion

Flatfoot affects various angular parameters, yet no study has specifically investigated the impact of a surgical procedure for hindfoot correction in children, such as STA, on first ray-related radiological parameters. 

Previous investigations in adults have shown varying outcomes regarding radiographic changes in the first ray following deformity correction surgery [6,7]. Wang et al. examined patients with Adult Acquired Flatfoot Deformity (AAFD) who underwent soft tissue and bony procedures, including a medializing calcaneal osteotomy or Cotton osteotomy [7]. They hypothesized that, following AAFD surgery, a reduction in HVA may occur due to first metatarsal bone supination. However, a medializing calcaneal osteotomy may also potentially lead to HVA augmentation due to factors such as abductor hallucis weakening, while a Cotton osteotomy may result in medial ray elongation. Postoperative findings revealed an improvement in the tibial sesamoid position, yet an average 4-degree increase in HVA was observed. These findings suggest that while initial first metatarsal bone supination post-surgery may be linked to medial soft tissue reconstruction and medializing calcaneal osteotomy, it may also result in HVA elevation due to abductor hallucis weakening and medial ray elongation. Additionally, the complex multiplanar corrective effects of these procedures may not be fully captured by two-dimensional radiographic parameters, and soft tissue imbalance could contribute to HV deformity pathogenesis. Matsumoto [6] retrospectively analyzed Hallux alignment changes in 37 feet treated with double or triple arthrodesis of the hindfoot for AAFD. Unlike their counterparts, the authors reported a reduction in the HVA following flatfoot correction surgery.

Unlike Matsumoto’s findings, our study did not show significant changes in first ray-related angles following STA intervention in pediatric patients. However, subsequent analyses conducted on the outcomes revealed noteworthy findings warranting further reflection.

Intra-observer and inter-observer reliability demonstrated excellent values, comparable to those reported in previous studies [17]. No significant changes exceeding ± 3.60 degrees in IM angles and ± 5 degrees in HV angles were observed. This cutoff was chosen to minimize X-ray misclassification, ensuring a misclassification risk of less than 5% [16,17]. While this conservative approach may reduce statistical sensitivity for significance, we emphasize the importance of maintaining scientific rigor in such studies.

The absence of statistically significant gender differences in angular parameter variations aligns with findings from other studies, which have reported a slightly better improvement in girls. Similarly, we also observed a trend toward greater improvement in girls, although this trend was not statistically significant in our study [5].

Regarding the correlation between preoperative HVA and IMA values and post-intervention correction, lower initial angle values appear to be associated with a slight worsening post-operation, while higher preoperative angular values tend to improve outcomes. These values are observed in individuals within the normal range, and in no case have they led to an increase in HVA beyond pathological values. Conversely, preoperative HVA and IMA values close to the threshold of pathology tend to exhibit higher postoperative correction values. The observed worsening of HVA values may be attributed to hallux abductor shortening due to hindfoot inversion secondary to STA, thereby reducing the varus action of this muscle. However, this consequence seems to be less influential in cases of more pronounced deformity at the first ray level; in this scenario, the beneficial effect of first ray supination likely outweighs the de-tensioning effect on the hallux abductor.

Finally, it is essential not to overlook how even minor variations in radiographic projection orientation or patient positioning during the radiographic examination can influence the measurements obtained, regardless of the accuracy and reproducibility of the measurements taken. With this radiographic bias, due to purely geometrical reasons, larger angles are more affected, and therefore, they are more susceptible to influence. Hence, it is not impossible to assert that one of the reasons why larger preoperative angles are associated with greater variations between pre and postoperative states may partly be attributed to this factor. However, the fact that almost all patients with larger preoperative deformities experienced a reduction in angular parameters post-operation suggests a genuine improvement in deformity only for this subgroup of individuals. 

## 5. Limitations

The limitations of this study primarily stem from the relatively short follow-up period post-surgery, which is nonetheless consistent with similar studies by Wang and Matsumoto [6,7]. Additionally, after 6 months, patients were able to resume high-impact sports, suggesting that the clinical condition was sufficiently stabilized for a radiological evaluation. However, assessing the same angles with longer follow-up periods would be of interest. Furthermore, the study is constrained by its small sample size and the retrospective design. Despite conducting a post hoc power analysis to ensure an adequate sample size, limitations persist due to the focus on angular values and the absence of an evaluation of the sesamoid bones position. While the procedure was anticipated to correct first ray-related radiological deformities, no statistically significant differences were observed between the preoperative and postoperative measurements. However, a significant correlation was identified between preoperative deformity values and their postoperative changes, indicating that the intervention might primarily benefit patients with pathological values. Furthermore, minor changes in radiological projections may have a disproportionate impact on higher radiological deformities, posing a challenge to accurately assessing outcomes. While weight-bearing foot CT scans may offer a solution in a scientific context, their practicality in clinical settings remains debatable.

## 6. Conclusions

STA showed positive radiological outcomes for PFF treatment, while negligible changes in first ray-related angles were observed. The age subgroup analysis indicated similar trends regardless of operation timing. Higher angles tended to improve, while lower preoperative angles tended to worsen post-operation, despite all falling within non-pathological ranges. Further research is warranted to confirm this correlation.

## Figures and Tables

**Table 1:** Differences in angle values between preoperative and postoperative measurement.

	Mean	Median	Standard Deviation	Minimum	Maximum
Age	11.7	11.5	1.3	10	15
Meary difference	6.53	6.21	8.76	−7.1	28.4
Calcaneal pitch difference	2.76	0.4	7.92	−11.4	27.9
Costa Bartani difference	13.1	14.1	11.4	−6.8	33.7
Kite angle difference	3.94	4.18	5.76	−7.07	16.7
IMA preop	8.7	8.98	2.14	4.9	13.6
IMA difference	−0.994	−1.19	2.8	−9.11	4.9
HVA preop	13.6	11.6	10.8	1.3	47.6
HVA difference	−2.02	0.555	9.28	−30.9	6.99

**Table 2:** Statistics in mean differences between pre-and postoperative values of first ray-related angles.

	IMA	HVA
Mean difference	−0.99	2.00
Pooled standard deviation	2.8	9.28
Standard error of the difference	0.44	1.46
Critical value for the student’s *t*-test at a sample significance level	1.975	1.975
Confidence interval for the difference between means	−0.99 ± 0.87	2.00 ± 2.88
Critical value	±3.6	±5

## Data Availability

Data supporting the findings of this study are available from the corresponding author upon reasonable request due to privacy reason.
